# Sentinel lymph node biopsy in vulvar cancer: status, level of knowledge, and counseling in outpatient setting

**DOI:** 10.1007/s00404-020-05701-4

**Published:** 2020-07-18

**Authors:** Marlene Röttger, Hermann Hertel, Laura Kaukemüller, Lars Brodowski, Markus Flentje, Peter Hillemanns, Rüdiger Klapdor

**Affiliations:** 1 Department of Obstetrics and Gynecology, Hanover Medical School, Carl-Neuberg-Street 1, 30625 Hannover, Germany; 2 Department of Anaesthesiology and Intensive Care Medicine, Hannover Medical School, Hannover, Germany

**Keywords:** Vulvar cancer, Sentinel lymph node biopsy, Outpatient setting, Groin recurrence

## Abstract

**Purpose:**

Evaluating the counseling of patients with vulvar cancer in outpatient setting regarding the application of sentinel lymph node dissection (SLND), the selection of hospitals for further treatment, and level of knowledge.

**Methods:**

A questionnaire containing 29 questions about SLND in vulvar cancer was sent to gynecologists in Lower Saxony. The questionnaire contained multiple choice questions and open questions. The study was approved by the local ethics committee.

**Results:**

The median age of the 86 respondents was 54 (26–66) years. Most participants (83.1%) reported to only treat one to five patients with vulvar cancer per year. Interestingly, 70.5% of the gynecologists send their patients to university hospitals and 64.1% to hospitals offering maximum care, respectively. Of all, 32.7% replied that SLND was performed rarely or never in their patients. The gynecologists answered that only 36.7% of the patients are well informed about advantages and possible disadvantages of SLND. Most (84%) felt responsible to counsel patients on treatment decisions independently from or additionally to the hospital. Of all, 72% replied that they are not completely sure about the exact recurrence rates after SLND. Of notice, 66% believe that SLND for vulvar cancer is safe if applied in specialized centers and 92% stated that focusing treatment on specialized centers is required for best results.

**Conclusion:**

SLND for vulvar cancer is widely accepted and regularly recommended among gynecologists. Outpatient doctors report to send most patients to specialized centers. However, it appears that patients remain uninformed after counseling in the clinics and that there is a lack of detailed knowledge about risks and complication rates of groin treatment in the outpatient setting.

**Electronic supplementary material:**

The online version of this article (10.1007/s00404-020-05701-4) contains supplementary material, which is available to authorized users.

## Introduction

The clinical relevance of vulvar cancer grows because of its increasing incidence [1]. For patients with vulvar cancer affection of lymph node metastases represents the most important prognostic factor for survival [1, 2]. The radical lymph node dissection (LND) of the groin was used as the standard procedure for diagnosis and therapy of lymph node metastases. However, this method is accompanied by a very high morbidity affecting more than half of the patients. The quality of life of patients after LND is restricted by wound healing disorders, lymphedema, and damaged nerves [3].

Since only 25–35% of all patients have lymph node metastases at primary diagnosis, most patients suffer from complications without having any benefit from this procedure [4, 5]. To prevent overtreatment in early-stage vulvar cancer, sentinel lymph node dissection (SLND) has been established as an alternative to radical LND, especially because of its significantly lower morbidity [5, 6]. In the GROINSS-V-I study, a prospective observational study, SLND led to low groin recurrence rates (2.3%) and low morbidity, if applied using restrictive inclusion criteria and stringent protocols [5]. Multiple retrospective studies have been performed showing mixed results with groin recurrence rates for SLND ranging between 0 and 12% [7]. According to a large meta-analysis comparing LND with SLND, groin recurrence rates appeared to be similar only under optimal conditions (unifocal tumors < 4 cm, clinically non-suspicious nodes in the groin, specific infrastructure, human resource, appropriate techniques, and procedures) [7].

Large randomized controlled studies are missing which makes it difficult to draw reliable conclusions.

It has to be kept in mind that groin recurrences entail a very high mortality [8]. An adequate selection of patients and treatment in a specialized center appears to be essential for a safe application of SLND [5]. According to a previous survey among German hospitals, we know that SLND is widely accepted and used in Germany. Interestingly, in that survey 43.5% of the clinics reported that they did not include patients according to the national guidelines [9]. It is still unknown, how clinicians make the choice between SLND and LND for their patients.

According to the German guideline, SLND represents an alternative to LND and can be performed, if patients are well informed about possible advantages and risks of SLND [1]. Due to a lack of randomized controlled trials counseling of patients requires close cooperation between outpatient doctors and clinics as well as exact knowledge and differentiated interpretation of the published literature.

Our aim was to evaluate how gynecologists in outpatient setting think about SLND, counsel patients and select hospitals for further treatment of this rare disease. These are important facts for understanding how patients are counseled and how hospitals and outpatient doctors communicate. Thereby, it shall help to develop strategies to guarantee provision of optimal care for vulvar cancer patients in the future.

## Materials and methods

A questionnaire containing 29 questions about SLND for vulvar cancer was sent to gynecologists (300) working in outpatient setting in Lower Saxony, Germany. There were multiple choice questions and open questions about the use, knowledge, and personal meaning of SLND. There were no mandatory questions. The questionnaire was sent per email once and as a paper version. In addition, the questionnaire was available via survey monkey (SurveyMonkey Inc., San Mateo, CA, USA, de.surveymonkey.com). An invitation to enter the study was sent per email three times using publicly available registers. All questionnaires were returned anonymously. All replies were collected in a database and evaluated using Microsoft Excel 2010 (Microsoft Corp., Redmond, WA, USA). The questionnaire was validated by five experts. The study was approved by the local ethics committee.

## Results

**Fig. 1:**
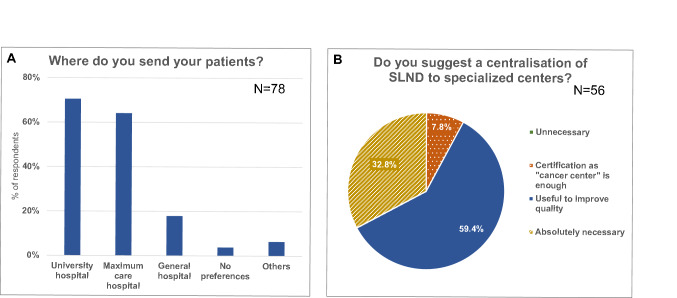
Type of hospitals patients are sent to for primary treatment (**a**) and evaluation of the necessity of centralization (**b**)

In the final evaluation 86 questionnaires were included (participation rate 28.7%). The general characteristics of the participating gynecologists are shown in Table 1. Cases of primary vulvar cancer were rare. Most of the participants (82.1%) reported to just treat 1–5 patients with primary vulvar cancer per year. The gynecologists send these patients to university hospitals and hospitals offering maximum care as shown in Fig. 1a. More than 90% of the participants recommend a centralization regarding the treatment of vulvar cancer patients in specialized centers (Fig. 1b).

**Table 1:** Characteristics of the participants

Characteristics	*N* (%)
Participants	86 (100%)
Male	19 (22.1%)
Female	65 (75.6%)
Self-employed	71 (82.6%)
Oncological specialization	15 (17.4%)

Vulvar cancer patients per year	*N* (%)
Respondents	77 (100%)
0	7 (7.8%)
1–5	64 (83.1%)
6–10	4 (5.2%)
11–30	3 (3.9%)

	Median (range)
Age	54 (26–66)
Years working in an outpatient setting	14 (0–31)

**Fig. 2:**
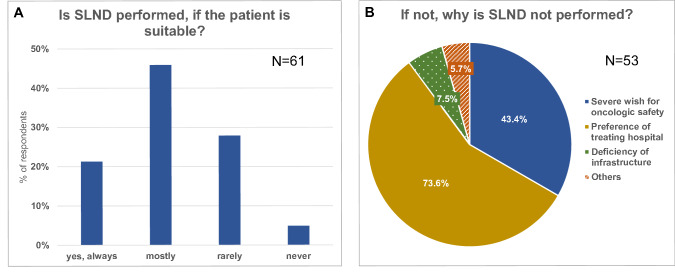
Do patients get SLND by default, if they are suitable (**a**) and why not (**b**)?

We asked the gynecologists, whether patients, who meet the criteria of the German guideline for SLND, get this treatment by default. Most of the participants (67.3%) replied “yes, always” or “in most cases”, but 32.7% answered “rarely” or “never” (Fig. 2a). The reasons given for this are shown in Fig. 2b. All in all, the SLND was rated positively: 66% judged the method to be “safe in specialized centers and under optimum conditions”.

The current communication between the gynecologists in outpatient setting and the treating hospital was generally valuated positively, but 32.4% of the participants judged the communication to be “moderate” or “bad”. As suggestions for improvement 63.6% of the participants demanded a faster and better feedback in the doctor’s letters and 35.1% asked for more information about current methods and treatments.

**Fig. 3:**
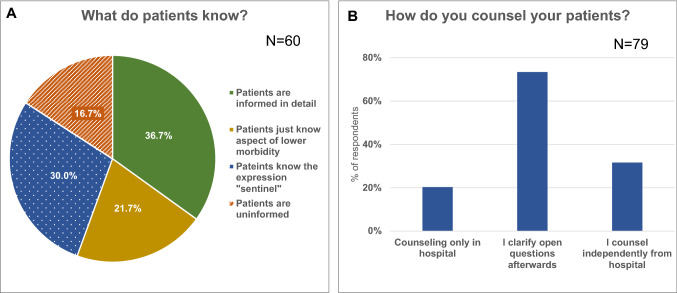
Knowledge of patients after medical briefing in hospital (**a**) and further counseling in outpatient setting (**b**)

According to the replies of the gynecologists, most of the patients do not seem to be adequately informed about the SLND after medical consultation in the treating hospital (Fig. 3a).

Accordingly, 73.4% of the participants report to clarify questions, which are still unanswered, after the briefing in the hospital and 31.6% counsel their patients completely independently from the treating hospital (Fig. 3b).

**Fig. 4:**
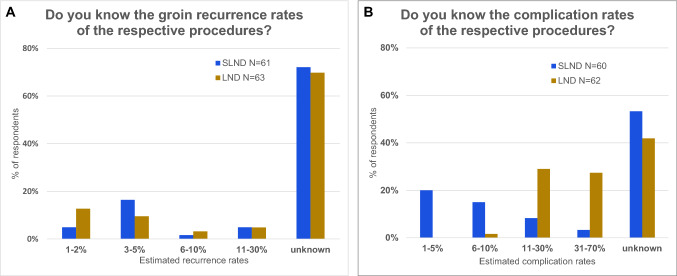
Knowledge about groin recurrence rates (**a**) and complications (**b**) after LND and SLND of the outpatient doctors

In order to learn more about the knowledge regarding SLND in outpatient counseling, we also included some questions about specific details. We asked for groin recurrence rates and complication rates especially, because these appear to be important facts for a profound counseling. Remarkably, about 70% of all participants replied to this difficult question and more than 70% of all gynecologists answered that they do not know specific groin recurrence rates for SLND or LND, respectively, as shown in Fig. 4.

We also compared whether gynecologists with specialization in oncology reported differently as compared to gynecologists without specialization. Interestingly, gynecologic oncologists reported that they send their patients more often to university hospitals (93.3% vs. 57.7%, *p* 0.09). We did not detect any differences regarding the value of SLND, counseling, or procedure-specific knowledge including recurrence rates and morbidity.

## Discussion

With this first study on the dealings with and the attitude towards SLND in outpatient setting in Germany, we could show that SLND for vulvar cancer is widely accepted and regularly recommended. Furthermore, in this survey we saw that outpatient doctors report to send most patients to specialized centers. However, it appears, that patients remain uninformed after counseling in the clinics and that there is a lack of detailed knowledge about risks and complication rates of groin treatment in the outpatient setting.

Although SLND has been widely introduced into the routine treatment of early-stage vulvar cancer [9], there are no large randomized controlled studies that prove a comparable oncological safety of SLND and radical groin dissection. The LACC study for cervical cancer has clearly shown, what impact randomized controlled studies may have for the final evaluation of a new treatment [10].

We have data from the prospective GROINS-V-I study (groin recurrence rate 2.3%) and some smaller studies (groin recurrence rates 0–12%), which show, that SLND, if applied under restrictive conditions, leads to low groin recurrence rates, which are comparable to those of historic collectives of groin LND [5, 7]. Similar results have been obtained by the subgroup analysis of the large retrospective multicenter AGO-CaRE-1 study [11]. According to the meta-analysis by Covens et al. the isolated groin recurrence rate of SLND (3.4%, 95% CI 1.8–5.4%) and LND (1.4%, 95% CI 0.4–2.9%) did not differ significantly [7]. However, there might be a tendency to worse results in the SLND group. However, final conclusions cannot be drawn since existing comparable studies do not have enough power to detect small differences.

We already know that groin recurrences after SLND are associated with a very high mortality (67–100%) [8]. Interestingly, according to Farrell et al. 80% of patients, already treated with LND, would choose the high risks of complications of LND over the benefits of SLND, if the risk of missing a positive lymph node was just higher than 1% [12]. These facts imply, that even if the groin recurrence rate after SLND might only slightly be elevated, patients have to be informed in detail in order to be able to make a profound and individual decision.

In this, the collaboration of doctors in hospitals and in outpatient setting has a huge importance, because vulvar cancer is a rare disease, which the treating doctors are not confronted with in their daily routine.

According to this study SLND is already used in most cases. Interestingly, 32.8% of the participants of our survey replied, that the method is not used, even though the patient was suitable for it according to German guidelines. Ideally, these would be patients, who were extensively informed about the method, and then decided to choose the radical LND instead, for example because of a severe wish for safety. This is what the guideline proposes. The patients should be counseled openly and unbiased [1]. According to the gynecologists in this study, in 73.6% of the cases the treating hospital was responsible for the choice of treatment.

Almost two-thirds of the patients are, according to our survey, not informed about the SLND sufficiently enough to make a sound decision after counseling in hospital. We cannot tell if this is related to the quality or extent of the medical briefing in hospital or if this reflects the normal situation after just one counseling appointment. However, it can be deduced that there are necessities for improving this situation. This also shows the importance of further counseling in outpatient setting afterwards. In order to improve the information of patients we believe that besides close cooperation with outpatient gynecologists information brochures and an additional preoperative counseling appointment in the hospital represent promising options.

Which information are needed to counsel a patient about the treatment of groin lymph nodes in vulvar cancer adequately? Among other things the oncologic safety of the method is definitely fundamental. We asked about some facts about LND and SLND (cf. Fig. 4). These questions turned out to be hard to answer for the participants. Certainly, this can be led back to the low incidence of the disease. But it is also a difficult question, because, as described above, randomized controlled trials are missing. The German guideline is mainly based on the GROINNS-V-I study, which declares the SLND to be an alternative to LND if applied under strict conditions (Table 2) [1, 5]. Since patients should be capable of choosing the right treatment for themselves, they have to be detailed informed about complication rates and groin recurrence rates.

**Table 2:** Requirements for using SLND in vulvar cancer according to the German Guidelines [1]

The following requirements should be met for using SLND in vulvar cancer
The diameter of the primary tumor is no larger than 4 cm in the vulvar plane
Unifocal tumor
No clinically and possibly sonographically suspicious groin lymph nodes
Experienced team in marking sentinel lymph nodes
Pathologic ultrastaging of the sentinel lymph nodes with additional immunohistochemical tests
Detailed counseling of the patient concerning advantages and possible oncological risks of this technique
Compliance of the patient for regular follow-up treatment

In the context of statistical literacy, several authors emphasized, that absolute risks and natural numbers are important tools to improve the understanding of the patients [13]. Therefore, in such a rare disease as vulvar cancer, it would be useful to provide this information in a condensed form such as leaflets or short decision guidelines. As a result of this survey, we created a short flyer containing all the necessary facts, which appear to be important for a profound patient counseling according to this study. This flyer shall help the doctors to have all necessary facts ready whenever needed in order to provide the patients with detailed information. This flyer was sent to all participants after the study and is accessible in the Supplements.

Taking these facts into account, especially in such a rare disease, the collaboration between hospitals and gynecologists in outpatient setting becomes more important. Considering the high variability of published groin recurrence rates after SLND, it is essential for each hospital to analyze and to discuss the own results of groin treatment and to include these results into the counseling of patients.

Owing to the scarcity of the disease, it appears to be important to centralize the treatment on specialized centers. This was supported by 92% of our participants and also corresponds to the German guideline [1].

We have to admit some restrictions to this analysis. Since this study is based on a facultative, open survey, we cannot rule out selection bias, meaning that more doctors preferring SLND over LND or otherwise participated in this study. However, with 80 gynecologists from Lower Saxony including specialists for gynecologic oncology and normal gynecologists this cohort seems to provide a good representation of the general situation. Nevertheless, this is the first study evaluating the attitude towards SLND in outpatient setting. The relevance of real-life data and health services research is strongly increasing. Therapeutic strategies can only be successful, if patients, outpatient doctors and hospitals closely collaborate. This study clearly helps to understand where communication and counseling can be improved to provide optimal results for the patients.

## Conclusion

All in all, there is a positive attitude towards SLND in outpatient setting. There already is good medical care for this rare disease, but of course there is uncertainty especially about the oncologic safety. A better and faster communication between hospital and gynecologists in outpatient setting is needed.

The treating hospital has a huge influence on the decision about the way of treating groin lymph nodes in vulvar cancer, but the individual counseling in outpatient setting is essential, just like the centralization of the treatment to a specialized center.

This study contains important information about the treatment of patients in outpatient setting and should be used to develop new structures for collaboration and improvement of care for patients with vulvar cancer.

## Electronic supplementary material

Below is the link to the electronic supplementary material.

Supplementary file1 (PDF 202 kb)

## Data Availability

Available on request.
