# Supplementary material for: Sentinel lymph node biopsy in vulvar cancer: status, level of knowledge, and counseling in outpatient setting
Source: Arch Gynecol Obstet. 2020 Jul 18;302(4):1001–7. doi: 10.1007/s00404-020-05701-4 (PMC7471199; doi:10.1007/s00404-020-05701-4)
Supplement: Supplementary file 1 — Supplementary file1 (PDF 202 kb) [file 404_2020_5701_MOESM1_ESM.pdf]

Ist die SLNE sicher?

Es gibt keine großen prospektiv randomisierten Studien!

GROINSS-I (4):
Prospektive einarmige, multizentrische Studie:
Unter optimalen Bedingungen in erfahrenen Zentren: niedrige isolierte Leistenrezidivraten bei SLN only (2.3%)

Eine Metaanalyse aus 2015 zeigt bei unifokalen Tumoren <4cm keinen signifikanten Unterschied für die Leistenrezidivrate (6):

Radikale LND: 1.4% (95%CI 0-3%)
SLND only: 3.4% (95%CI 2-4%) (5)

Keine eindeutige Klärung der Frage. Nach der größten prospektiven Studie (GROINSS-V) führt die SLNE zu niedrigen Leistenrezidivraten unter optimaler Patientenselektion in einem spezialisierten Zentrum (4)

Welche Risiken bestehen bei der SLNE?

- Falsch-Negativ-Rate sehr abhängig von Patienten-selektion, methodischer Durchführung und Erfahrung des Operateurs (1)
- Bei falsch negativen SLN erhöhte Leistenrezidivrate
- Leistenrezidiv geht mit sehr hoher Mortalität (67-100%) einher (3)

Quellen:

(1)Diagnosis, Therapy and Follow-Up Care of Vulvar cancer and its Precursors National Guideline of the German Society of Gynecology and Obstetrics (S2k-Level, AWMF Registry No. 015/059, August 2015). http://www.awmf.org/leitlinien/detail/ll/015-059.html.

(2) Klapdor R, Hertel H, Soergel P, Jentschke M, Hillemanns P. Application of sentinel lymph node dissection in gynecological cancers: results of a survey among German hospitals. Arch Gynecol Obstet. 2017 Mar;295(3):713-720. doi: 10.1007/s00404-016-4279-3

(3) Klapdor R, Hertel H, Soergel P, Hillemanns P. Groin Recurrences in Node Negative Vulvar Cancer Patients After Sole Sentinel Lymph Node Dissection. Int J Gynecol Cancer. 2017 Jan;27(1):166-170. doi: 10.1097/IGC.0000000000000860

(4)Van der Zee, Ate G J, Oonk MH, De Hullu, Joanne A, et al. Sentinel node dissection is safe in the treatment of early-stage vulvar cancer. J Clin Oncol. 2008;26(6):884-889. doi:10.1200/JCO.2007.14.0566.

(5) Covens A, Vella ET, Kennedy EB, Reade CJ, Jimenez W, Le T. Sentinel lymph node biopsy in vulvar cancer: Systematic review, meta-analysis and guideline recommendations. Gynecol Oncol. 2015 May;137(2):351-61. doi: 10.1016/j.ygyno.2015.02.014

Medizinische Hochschule Hannover

Sentinellymphonodektomie beim Vulvakarzinom

Leitfaden zur Patientenberatung

Einleitung

Die Lymphknotenmetastasierung stellt beim Vulvakarzinom den wichtigsten prognostischen Faktor für das Überleben dar (1). Zur Diagnostik und Therapie von Lymphknotenmetastasen dient die radikale inguino-femorale Lymphonodektomie. Diese Methode geht aber mit einer sehr hohen Morbidität einher:

Wundheilungsstörungen 14-44%
Lymphozelen 13-40%
Lymphödem (therapiepflichtig) 20-35% (1)

Da nur in 25-35% tatsächlich Lymphknotenmetastasen vorliegen, wird die Sentinel-lymphonodektomie (SLNE) zunehmend als Alternative angewendet, da sie mit einer deutlich geringeren Morbidität einhergeht (2).
Da ein Leistenrezidiv aber, z.B. aufgrund fehlerhafter Technik oder falscher Indikation, mit einer sehr hohen Mortalität der Patientin verbunden ist, erscheint es entscheidend, eine adäquate Patientenselektion für dieses Verfahren durchzuführen (3).

Wir möchten Ihnen in diesem Flyer die wichtigsten Informationen zur Beratung der Patientinnen mit Vulvakarzinom aus der aktuellen Literatur zusammenstellen.

Ab wann werden die Lymphknoten beim Vulvakarzinom untersucht?

Ab einer Infiltrationstiefe von mehr **als 1mm (≥pT1b)** soll ein operatives Staging der inguino-femoralen Lymphknoten durchgeführt werden (1).

Voraussetzungen für eine Sentinel-Lymphknoten-Biopsie nach (1):

- Maximaler Tumordurchmesser in der Hautebene <4cm
- Unifokaler Tumor
- Klinisch und ggf. sonographisch unauffällige Leistenlymphknoten

- Erfahrung im Team mit dem Sentinel-markierungsverfahren
- Ultrastaging der Lymphknoten mit immunhistochemischer Untersuchung durch den Pathologen
- Eingehende Aufklärung der Patientin über Vorteile und mögliche onkologische Risiken der Methode
- Compliance der Patientin für eine regelmäßige Nachsorge

Empfehlung zur SLNE in der Leitlinie?

Bei Erfüllung der Kriterien soll die Patientin über die Vorteile und auch die möglichen onkologischen Risiken der Sentinel-lymphonodektomie und der systematischen inguino-femoralen Lymphonodektomie aufgeklärt werden.
Wird eine Sentinellymphonodektomie nicht durchgeführt, soll die inguino-femorale Lymphonodektomie erfolgen (1).

Wofür Ultrastaging?

Ultrastaging bezeichnet eine gezielte histologische Aufarbeitung der Lymphknoten durch z.B. engmaschigere Schnittebenen und immun-histochemische Aufarbeitung. Hierdurch steigt die Wahrscheinlichkeit Mikrometastasen zu entdecken um 12-40%. Diese Mikrometastasen haben prognostische Bedeutung. Daher ist das Ultrastaging entscheidend für eine sichere Anwendung der SLNE und sollte Grundvoraussetzung sein, um das Verfahren anbieten zu können. (1).

Wird die SLNE in Deutschland durchgeführt?

Nach einer deutschlandweiten Umfrage 2016 führen 73% der antwortenden Kliniken eine SLNE in Deutschland durch. Im Mittel werden 7.8 (1-43) SLNE pro Jahr durchgeführt. Nur 56.5% der Kliniken gaben an, die Patientinnen analog der Leitlinien zu behandeln und ein Ultrastaging der Lymphknoten durchzuführen. Insgesamt gaben 77.4% der Kliniken an, dass sie die SLNE beim Vulvakarzinom als sicher erachten (2).
